# Attenuated expression of SNF5 facilitates progression of bladder cancer via STAT3 activation

**DOI:** 10.1186/s12935-021-02363-3

**Published:** 2021-12-07

**Authors:** Hua Ding, Yaqin Huang, Jiazhong Shi, Liwei Wang, Sha Liu, Baixiong Zhao, Yuting Liu, Jin Yang, Zhiwen Chen

**Affiliations:** 1 Department of Urology, Southwest Hospital, Third Military Medical University (Army Medical University), Chongqing, 400038 China; 2 Department of Cell Biology, Third Military Medical University (Army Medical University), Chongqing, 400038 China; 3 Unit 32357 of People’s Liberation Army, Pujiang, 611630 China

**Keywords:** SNF5, Bladder cancer, STAT3, Individualized treatment

## Abstract

**Background:**

SWI/SNF, a well-known ATP-dependent chromatin-remodeling complex, plays an essential role in several biological processes. SNF5, the core subunit of the SWI/SNF remodeling complex, inactivated in 95% of malignant rhabdoid tumors (MRT), highlighting its significance in tumorigenesis. However, the role of SNF5 in bladder cancer (BC) remains unknown. In this study, we aimed to investigate the function and potential clinical applicability of SNF5 in BC.

**Methods:**

Data from The Cancer Genome Atlas (TCGA), Gene Expression Omnibus (GEO) and Cancer Cell Line Encyclopedia (CCLE) databases were used to evaluate the clinical significance of SNF5 in BC. We performed Gene Set Enrichment Analysis (GSEA) and functional assays to investigate the role of SNF5 in BC. Genomics of Drug Sensitivity in Cancer (GDSC) and drug-susceptibility tests were performed to identify the potential value of SNF5 in the treatment of BC.

**Results:**

Low SNF5 expression conferred a poor prognosis and was significantly associated with the N-stage in BC. ROC curves indicated that SNF5 could distinguish BC from the normal tissues. In vitro and in vivo functional assays demonstrated that attenuated SNF5 expression could promote cell proliferation and enhance migration by STAT3 activation. We imputed that low SNF5 expression could confer greater resistance against conventional first-line drugs, including cisplatin and gemcitabine in BC. GDSC and drug-resistance assays suggested that low SNF5 expression renders T24 and 5637 cells high sensitivity to EGFR inhibitor gefitinib, and combination of EZH2 inhibitor GSK126 and cisplatin.

**Conclusions:**

To the best of our knowledge, the present study, for the first time, showed that low SNF5 expression could promote cell proliferation and migration by activating STAT3 and confer poor prognosis in BC. Importantly, SNF5 expression may be a promising candidate for identifying BC patients who could benefit from EGFR-targeted chemotherapy or cisplatin in combination with EZH2 inhibitor treatment regimens.

**Supplementary Information:**

The online version contains supplementary material available at 10.1186/s12935-021-02363-3.

## Background

The global cancer statistics showed that bladder cancer (BC) caused an estimated 573,000 new cases and 213,000 deaths in the year 2020 [1]. Traditionally, BC is categorized into non–muscle-invasive bladder cancer (NMIBC), which is characterized by a high recurrence rate, and muscle-invasive bladder cancer (MIBC), which is prone to metastasis and has a poor prognosis [2, 3]. The 5-year survival rate of BC has not improved over the past three decades [4]. Therefore, it is essential to understand the molecular mechanisms that underlie BC tumorigenesis and progression to develop more effective therapeutic strategies.

The SWI/SNF complex was first discovered and isolated from yeast. It is highly conserved across the eukaryotic kingdoms [5]. Several previous studies show that researches have shown that in normal tissues, the SWI/SNF complex can functionally regulate genes associated with DNA repair, cell cycle, and cell division [6]. The subunits of the SWI/SNF complex are frequently dysregulated in 25% of all carcinomas [7], which underscores the significance of the SWI/SNF complex in carcinogenesis.

SNF5 (also known as SMARCB1) encodes a ~ 50 kDa protein, which is the core subunit of the SWI/SNF complex [8]. Approximately 95% of the malignant rhabdoid tumors (MRT) harbor aberrations in the SNF5 subunit [9, 10]. In vivo experiments using mouse models demonstrate that SNF5 loss results in rapid formation of tumors in all subjects at 11 weeks, which is half the time taken for TP53-induced tumorigenesis [11, 12]. Several studies focused on understanding the SNF5-mediated suppression of tumorigenesis. Recently, a mechanistic study demonstrates that SNF5 antagonizes MYC, an oncoprotein transcription factor, by impairing its DNA-binding ability in MRT; SNF5 inactivation can synergistically accelerate tumor formation in combination with p53 loss [13]. Similarly, in myeloid leukemia, SNF5 downregulation induces the activation of Rac GTPase, thereby promoting cell migration and survival [14]. Given the abovementioned findings, SNF5 has long been considered a tumor suppressor. Nevertheless, SNF5 is upregulated in liver cancer and seems to play an oncogenic role [15], suggestive of its dichotomy in tumorigenesis. To date, however, the effects of SNF5 on BC and the underlying mechanisms remain unknown.

Thus, this study aimed to investigate the functions of SNF5 and the mechanisms underlying SNF5 mediated pathological features in BC. Furthermore, the clinical potential of SNF5 in BC was evaluated by using public database and drug susceptibility tests. The findings may facilitate individualized clinical management of BC patients.

## Methods

### Cell culture and transfection

The T24, 5637, and UM-UC-3 human BC cell lines and the normal urothelial cell line SV-HUC-1 were purchased from the Cell Bank of the Chinese Academy of Science (Shanghai, China). The cells were cultured in RPMI-1640 (HyClone, USA) or MEM medium (HyClone, USA) supplemented with 10% fetal bovine serum (FBS) (Gibco, USA) at 37 °C in 5% CO2. Lentiviruses for SNF5 knockdown and SNF5 overexpression were synthesized by GenePharma Co. Ltd. (Shanghai, China), and lentiviral transduction was performed according to the manufacturer’s instructions.

### CCK-8 assay

CCK-8 assay was used for cell proliferation and drug susceptibility tests. Drugs used were AKT inhibitor VIII (HY-10355), gefitinib (HY-50895) and AZD-0530 (HY-10234), were purchased from MedChem Express (Monmouth Junction, NJ, USA). The cells were seeded in 96-well plates (800 or 5000 cells/well) and cultured for 4 days or 3 days. Cell viability was evaluated using the Cell Counting Kit 8 (CCK8; Dojindo, Japan) according to the manufacturer’s instructions. Briefly, 10 µL CCK-8 solution was added to each well at pre-determined time points, and the cells were incubated for 2 h. Absorbance at 450 nm was detected using a microplate reader (Bio-Rad, USA).

### Colony-formation assay

As described previously [16], the cells were seeded in 6-well plates (800 cells/well) and cultured for approximately 14 days. The colonies thus obtained were washed and fixed with 4% paraformaldehyde, stained with crystal violet for 20 min, and air-dried after rinsing off the excess dye. The number of colonies that were visible to the naked eye was counted.

### Wound-healing assay

The wound-healing assay was performed using the Culture-Inserts 2 Well system (Ibidi GmbH, Germany). Serum-starved cells were seeded into the inserts. The images were acquired at predetermined time points after the removal of the culture inserts. The cell migratory ability was calculated as follows:

Wound-healing percentage = (Area of the original scratch−Area of the scratch at pre-determined timepoint)/Area of the original scratch × 100. The Image J software (version 1.52, NIH, USA) was used to analyze the area.

### Transwell chamber assay

Cell migration was also investigated using the Transwell chamber assay (Corning, USA), according to the manufacturer’s instructions. Briefly, serum-starved cells were resuspended in 0.2 mL serum-free medium and transferred into the upper chamber. In the lower chamber, 0.6 mL RPMI-1640 medium supplemented with 10% FBS was added as a chemoattractant. The cells on the upper surface of the chamber were scrubbed and those on the lower surfaces were fixed with 4% paraformaldehyde and stained with crystal violet. These cells were photographed, and counted in five random fields.

### Tumorigenicity assays

An in vivo tumor xenograft model was established using five-week-old nude mice purchased from the Third Military Medical University (Chongqing, China). An equal number of T24 or 5637 live cells were injected subcutaneously into the root of the right thigh for each mouse after counting and excluding the trypan blue-stained cells. The tumor size was measured using vernier calipers and the tumor volume was calculated as follows:

Volume (mm3) = (length × width^2^)/2. The mice were euthanized intraperitoneally by injecting an overdose of pentobarbital sodium (150 mg/kg). Finally, the end-point tumors were excised.

### Western blotting

Total protein was extracted from the cells using the RIPA lysis buffer (Beyotime Biotechnology, Shanghai, China), and measured using a BCA kit (Beyotime Biotechnology, Shanghai, China). Equal amounts of protein per sample were diluted in 5X SDS loading buffer, separated in 10% SDS-PAGE gel, and transferred to a PVDF membrane. The blots were incubated sequentially with the primary and corresponding secondary antibodies. The primary antibodies included SNF5 (CST, 91735S; 1:1000), Snail (CST, 3879S; 1:1000), E-cadherin (CST, 14472; 1:1000), Vimentin (Bioworld, 1491; 1:1000), GAPDH (Affinity, AF7021; 1:3000), signal transducer and activator of transcription (STAT3; CST, 9139S; 1:1000), and pSTAT3-Y705 (CST, 9145S; 1:1000). The protein bands were visualized using the ECL reagent (Bio-Rad, CA, United States), quantified by Image J (National Institutes of Health, USA) and the relative protein levels were normalized to that of GAPDH.

### Immunohistochemistry (IHC)

IHC was performed as previously described [17]. Briefly, tissues were fixed, paraffin-embedded, and sliced into 5 μm thick sections. The protein expression of SNF5 (CST, 91735, 1:1000), pSTAT3-Y705 (CST, 9145, 1:400), Ki67 (CST, 9027,1:500), and cleaved caspase 3 (CST, 9661,1:400) was detected and scored according to the above-mentioned reference.

### Bioinformatics analysis

The transcriptomic and clinicopathological data of BC patients were obtained from TCGA using UCSC Xena. GSE13507 was downloaded from the GEO database and normalized by the RMA package [18]. The patient characteristics are summarized in Additional file 1: Tables S1 and Additional file 2: Tables S2. The gene set enrichment analysis (GSEA) was performed with GSEA-4.0 software (http://software.broad institute.org/gsea/). The survival probability was evaluated with the Kaplan–Meier method, and the differences were determined by the log-rank test (https://CRAN.R-project.org/package=survminer). The pROC package was used for receiver operating characteristic (ROC) analysis [19]. Using the GDSC, we estimated the chemotherapeutic sensitivity of patients in the TCGA data set. The prediction process was conducted by the pRRophetic package [20]. The half-maximal inhibitory concentration (IC_50_) for each sample was calculated by ridge regression via tenfold cross-validation using the GDSC training set.

### Statistical analyses

Statistical analyses were performed using Prism 8.0 and R software (version 4.0). All data were presented as mean ± SD, and the intergroup differences were analyzed using the two-tailed Student’s t-test. The differences among multiple groups were assessed by analysis of variance (ANOVA). P < 0.05 was considered statistically significant.

## Results

### Low SNF5 expression confers poor outcome in BC patients

**Fig. 1:**
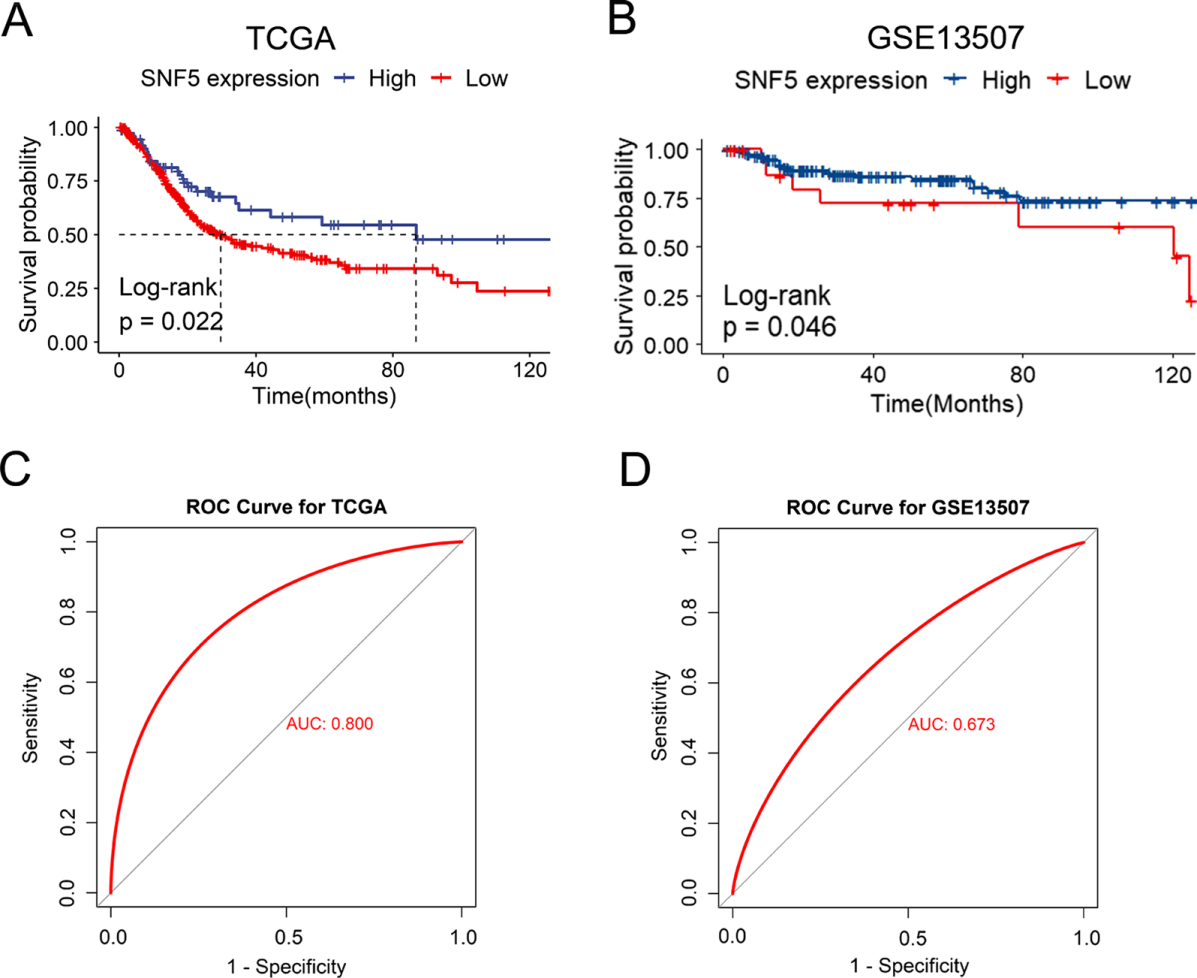
Low SNF5 expression confers poor outcomes in BC patients. **A** The Kaplan–Meier analysis of overall survival in TCGA database and **B** GSE13507 cohort. ROC curves for **C** TCGA and **D** GSE13507 cohorts

**Fig. 2:**
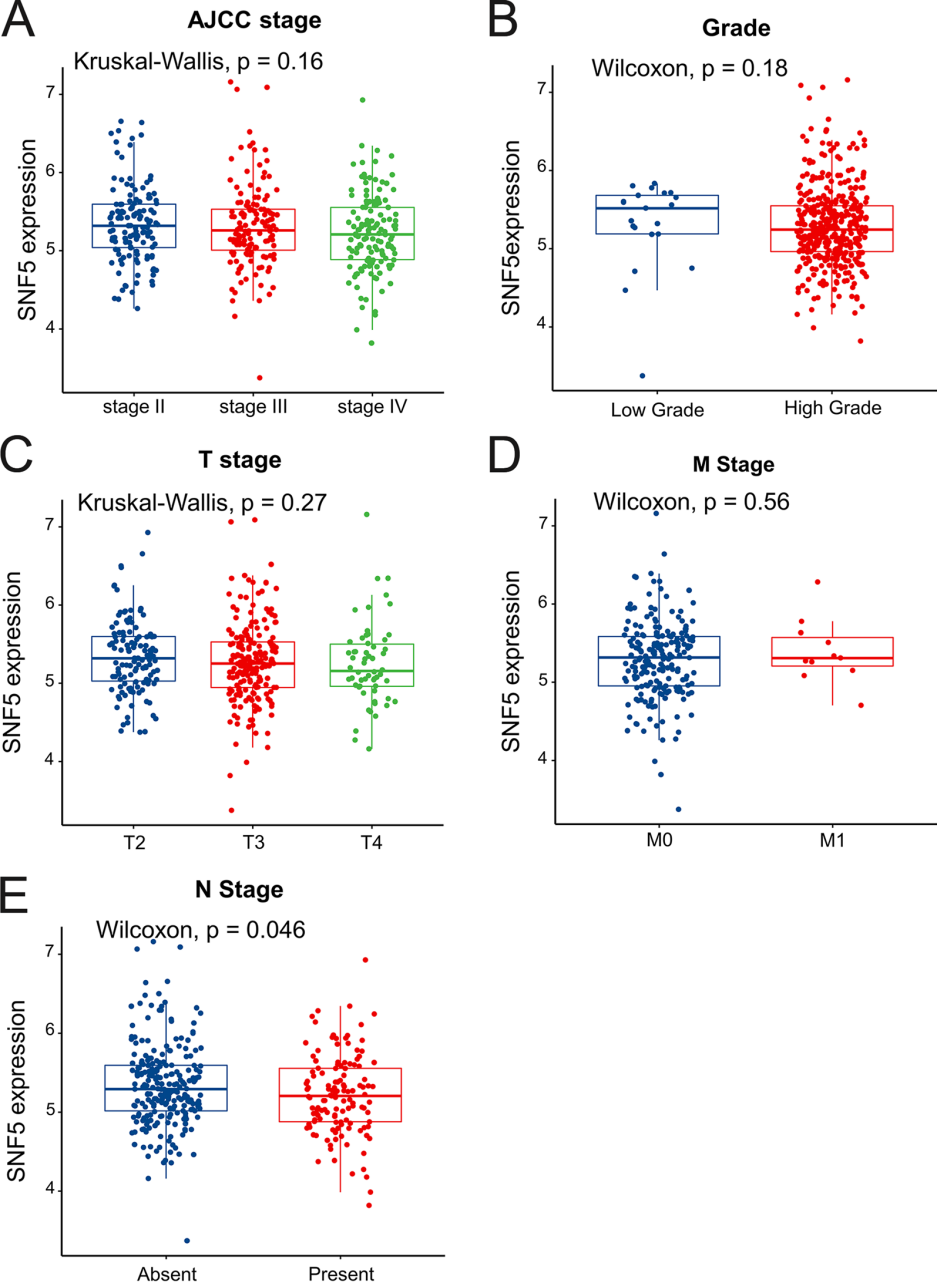
Significance of SNF5 in clinicopathological features of BC. SNF5 expression in different **A** AJCC stages, **B** Grades, **C** T stages, **D** M stages, and **E** N stages in BC

To investigate whether SNF5 was associated with the clinical outcomes of BC patients, a survival analysis was performed based on the data from TCGA database. Patients with low SNF5 expression were found to have significantly worse outcomes than those with high SNF5 expression (Fig. 1A). A significantly shorter survival time was consistent among patients with low SNF5 expression as compared to those with high SNF5 expression in the GSE13507 cohort (Fig. 1B). To assess the potential role of SNF5 in predicting BC, the ROC curve analysis was performed for patients in TCGA and GSE13507 datasets. The area under the curves (AUC) were 0.8 and 0.673, respectively, which suggested the favorable performance of SNF5 in distinguishing BC from normal tissues (Fig. 1C, D). Given the close association of SNF5 with prognosis, we further explored the relationship between SNF5 expression and the clinical characteristics of BC in TCGA, which comprises a larger sample size and includes more comprehensive information of the enrolled patients. We found no significant associations between clinical characteristics with SNF5 expression (Additional file 3: Tables S3). Consistently, SNF5 expression did not differ significantly among different grades, AJCC stages, T stages, or M stages (Fig. 2A–D). Notably, SNF5 expression showed a decreasing trend in the more advanced AJCC stages and grades, albeit the findings were statistically insignificant. Importantly, patients with lymphatic metastasis were significantly associated with lower SNF5 expression as compared to those without lymphatic metastasis (Fig. 2E).

**Fig. 3:**
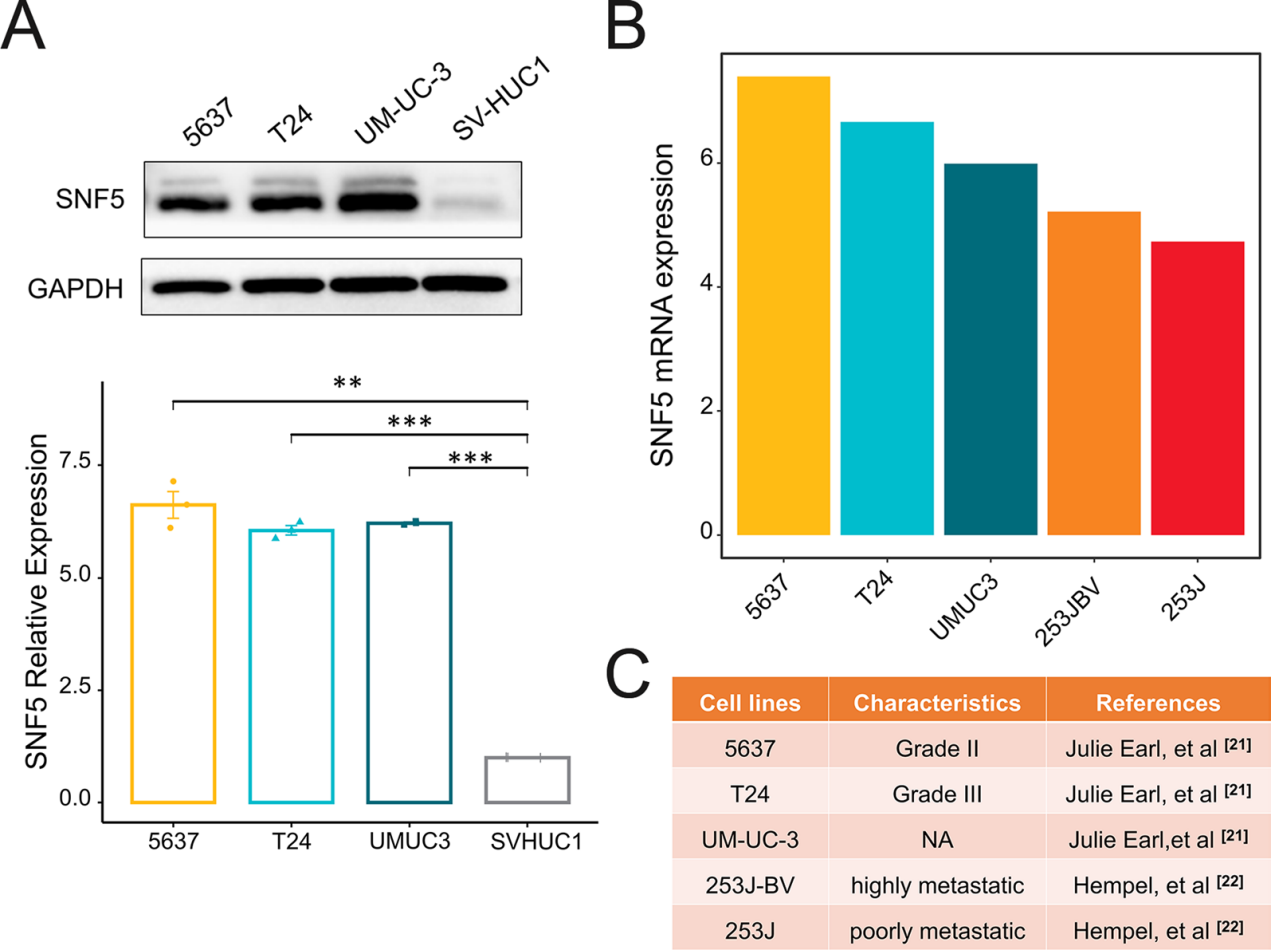
SNF5 expression in cell lines. **A** The SNF5 expressions in cell lines were detected by western blotting. The quantitative analysis of western blots was normalized by expression SV-HUC1. **B** Analysis of SNF5 mRNA expression in CCLE database. **C** Information on BC cell lines. *p < 0.05, **p < 0.01, ***p < 0.001

Next, the protein level expression of SNF5 was verified in the cell lines. BC cell lines, including 5637, T24, and UM-UC-3, demonstrated higher SNF5 expression than normal urothelial cells (i.e., SV-HUC1; Fig. 3A). T24 cells have been derived from a more advanced tumor grade than are 5637 cells [21], which showed the highest SNF5 expression among the BC cell lines. Additionally, we examined the expression of SNF5 in the Cancer Cell Line Encyclopedia (CCLE) database. Two metastatic BC cell lines, 253 J and 253 J-BV [22] had the lowest SNF5 expression (Fig. 3B–C). Combined with the above-mentioned data from the public database, we inferred that low SNF5 expression was significantly associated with metastasis.

### SNF5 downregulation facilitates BC cell proliferation in vitro and in vivo

**Fig. 4:**
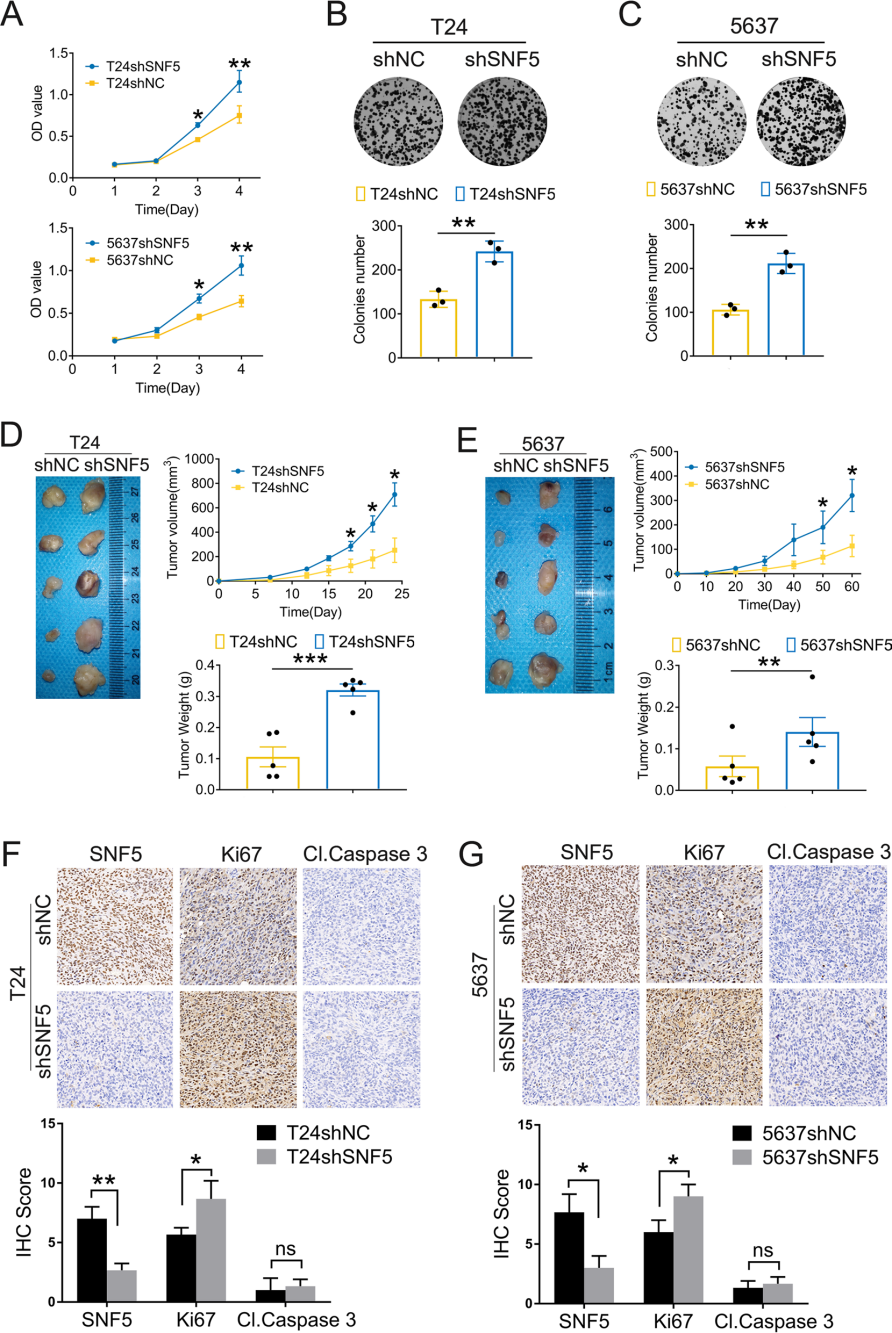
SNF5 depletion promotes cell proliferation in vitro and in vivo. **A** CCK8 assay shows that reduced SNF5 facilitates the proliferation of both T24 and 5637 cells. **B**–**C** Colony formation assay was performed to evaluate cloning abilities SNF5-depleted or control BC cells. **D**–**E** Images of the xenograft tumors from nude mice with subcutaneously injected with SNF5-depleted or negative control BC cells. Tumor growth curves were plotted and tumor weights were compared in shNC group and shSNF5 group. **F**–**G** Immunohistochemistry assay of SNF5, Ki67 and Cleaved Caspase 3 expressions in tumor xenografts in each group of nude mice. *p < 0.05, **p < 0.01, ***p < 0.001

To explore the biological functions of SNF5 in BC, we knocked down SNF5 in T24 and 5637 cells, and overexpressed SNF5 in T24 cells. First, the efficiency was confirmed by western blotting (Additional file 4: Fig. S1, Additional file 5: Fig. S2A). Owing to its higher knockdown efficiency, we chose shSNF5-2# for the subsequent experiments. The CCK8 assay indicated that a decrease in SNF5 expression could promote cell proliferation in T24 and 5637 cells (Fig. 4A). The results of the colony-formation assay further confirmed that SNF5 knockdown could facilitate cell proliferation in both the T24 and 5637 cells (Fig. 4B, C). However, overexpression of SNF5 in T24 cells had no significant effect on cell proliferation (Additional file 5: Fig.S2B).

Based on the in vitro results, the pro-proliferative effect of SNF5 depletion was determined in tumor xenograft models by subcutaneously injecting BC cells carrying SNF5 knockdown (shSNF5) or the corresponding negative control (shNC) vectors. The tumor growth rate was significantly higher and tumor weights remarkably increased in the T24 SNF5- depleted group than those in the control group (Fig. 4D). Likewise, the growth of xenografts from SNF5-depleted 5637 cells grew rapidly and weighed significantly greater than those formed by 5637shNC cells (Fig. 4E). Moreover, IHC staining showed that tumor tissues derived from the T24- and 5637 SNF5-depleted cells had higher levels of Ki67 as compared to that in the shNC group (Fig. 4F, G). However, no significant difference was observed in the expression of cleaved caspase 3. Thus, attenuated SNF5 expression conferred a positive effect on BC tumorigenesis.

### Attenuation of SNF5 enhances cell migration in BC

**Fig. 5:**
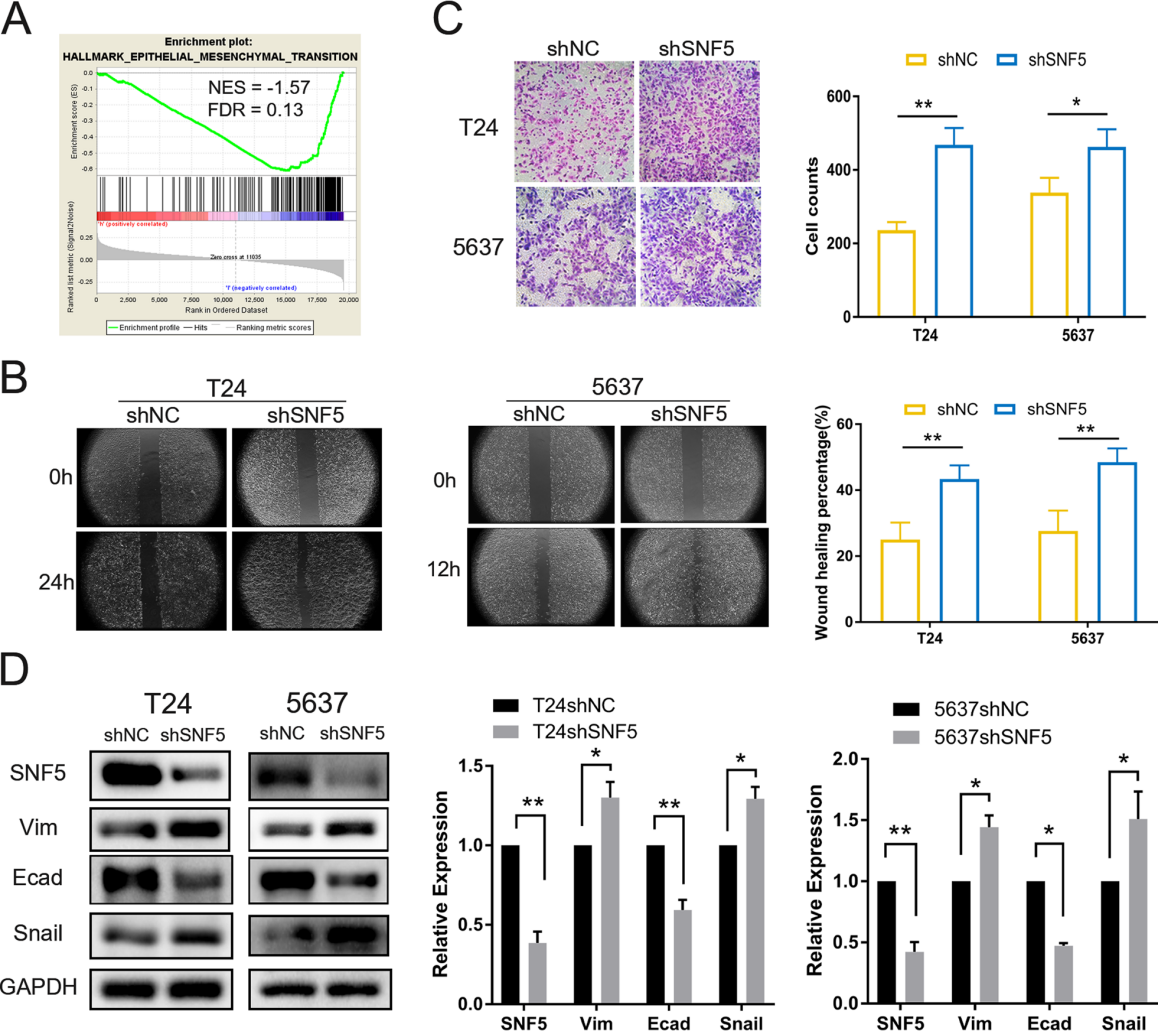
Knocking down SNF5 accelerates cell migration in BC. **A** GSEA analysis shows that EMT-related gene sets are enriched in the SNF5 low expression group in TCGA. **B** Wound healing assay was performed to determine the migration ability of T24 and 5637 after the stable knockdown of SNF5 (100X). **C** Transwell assays using SNF knockdown (shSNF5) and negative control (shNC) cells performed to assess their migration ability (200X). **D** Western blotting was used to detect the expression of EMT related markers (Vimentin, E-cadherin and Snail) in the indicated cells. *p < 0.05, **p < 0.01

To examine whether SNF5 was involved in cell migration, GSEA was performed. GSEA was based on the RNA-seq data of clinical samples from the TCGA-BLCA cohort and the results showed that the epithelial-mesenchymal transition (EMT) gene sets were significantly enriched in the SNF5 low-expression group, which indicating that SNF5 could play a role in EMT (Fig. 5A). Next, the wound-healing assay was performed to evaluate the effect of SNF5 on cell migration. The migratory speeds of T24 and 5637 cells were significantly enhanced upon SNF5 depletion, as compared to their counterparts (Fig. 5B). The results of the Transwell migration assay were consistent as the enhanced migration abilities of T24 and 5637 SNF5 knockdown cells were observed (Fig. 5C). However, no significant effect on cell migration was observed in T24 cells having SNF5 overexpression (Additional file 5: Fig.S2C–D). Western blotting showed that vimentin and snail, two EMT signature molecules, were elevated in SNF5-depleted cells, which was accompanied with by an obvious downregulation of E-cadherin (Fig. 5D). Collectively, these data suggested that attenuation of SNF5 expression facilitated cell migration in BC cells.

### Depletion of SNF5 promotes BC progression by regulating STAT3

**Fig. 6:**
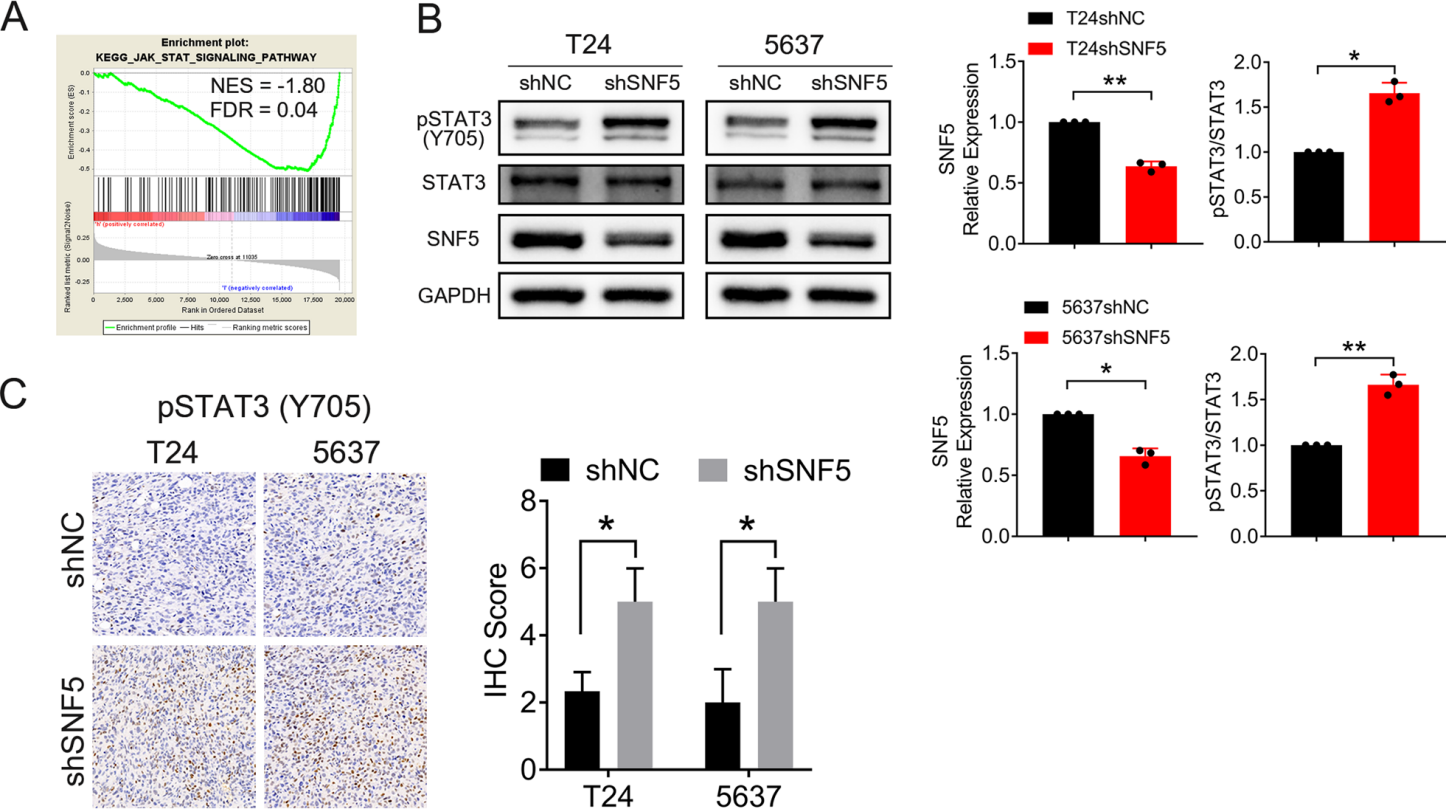
SNF5 depletion activates the STAT3 signaling pathway. **A** GSEA shows that the JAK/STAT signaling pathway is enriched in the low SNF5 expression group in TCGA. **B** Western blotting indicates that attenuated SNF5 activates STAT3. **C** IHC staining for p-STAT3 (Y705) in the indicated xenografts (200X). *p < 0.05, **p < 0.01, ***p < 0.001

To guide the identification of pathways critical for the aggressive BC phenotype upon SNF5 knockdown, GSEA was performed based on the canonical pathways from the KEGG database and the hallmark gene set collection [23, 24]. The results showed that genes involved in the JAK/STAT signaling pathway were significantly enriched in BC samples with low SNF5 expression (Fig. 6A). Emerging evidence indicates that STAT3 is a key oncogene which is implicated in the activation of the signaling pathways involved in cell proliferation and cancer metastasis [25, 26].

To investigate whether SNF5 affected STAT3 activation, western blotting was performed. We found that phosphorylated STAT3 (Y705) expression was significantly encreased in BC cells upon SNF5 depletion (Fig. 6B). Consistently, IHC staining confirmed the elevated expression of pSTAT3(Y705) in SNF5-depleted xenograft tumor tissues (Fig. 6C). These data showed that SNF5 depletion triggered STAT3 activation in BC cells.

**Fig. 7:**
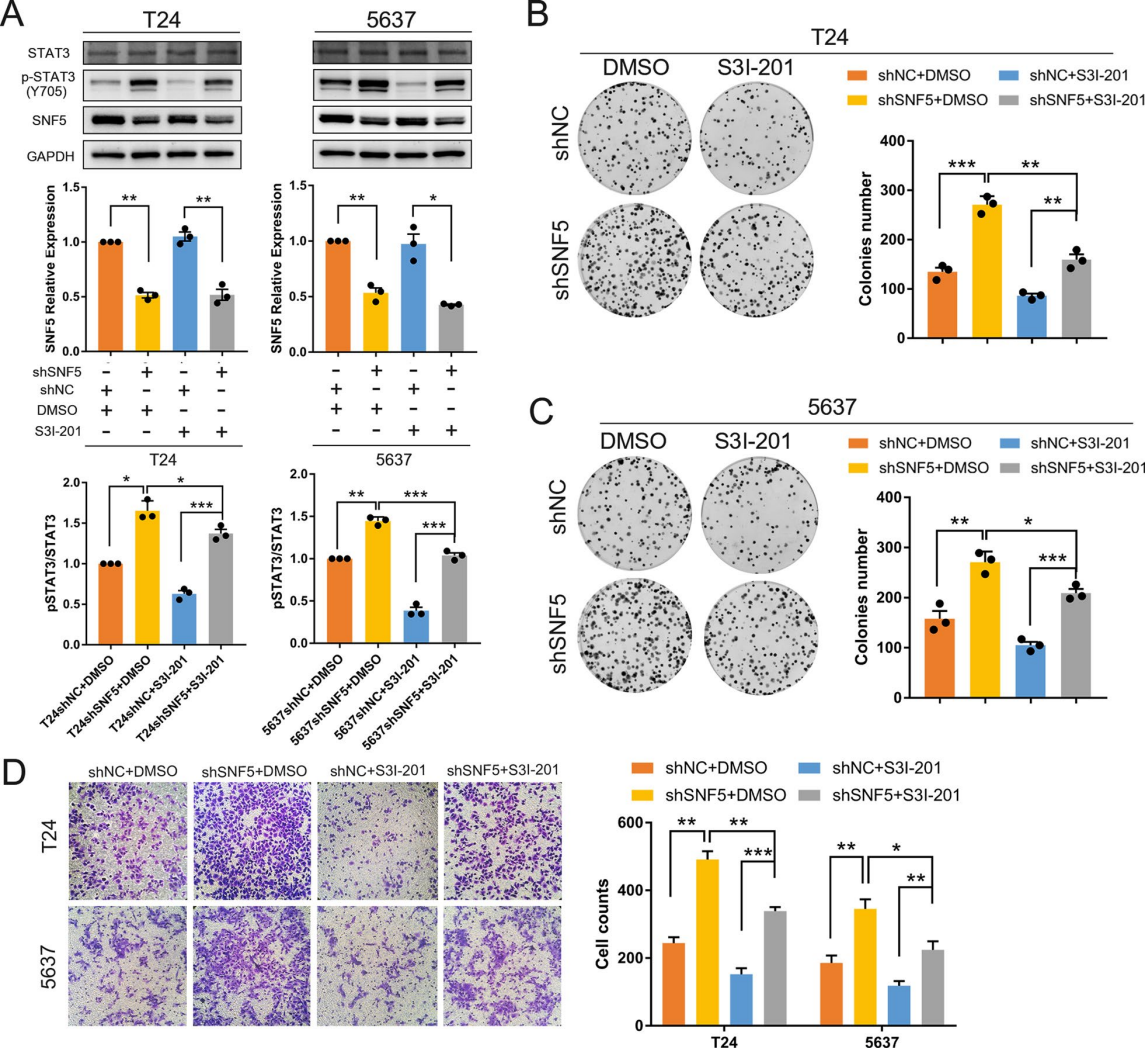
STAT3 inhibition suppresses the enhancing effect of SNF5 depletion in T24 and 5637 cells. **A** Western blotting shows that the expression of p-STAT3 (Y705) is compromised after treatment with S3I-201, a STAT3 inhibitor. **B**–**C** The colony formation assays and **D** Transwell assays show the suppressive effects of S3I-201 on enhanced cell proliferation and migration upon SNF5 knockdown in T24 and 5637 cells with (200X)

To verify whether the SNF5-induced enhanced effects were mediated by STAT3 activation, BC cells were treated with the STAT3 inhibitor, S3I-201. The expression levels of pSTAT3 (Y705) in BC cells were attenuated after treatment S3I-201 (Fig. 7A). Colony-formation assay indicated that inhibition of STAT3 signaling could significantly suppress cell proliferation (Fig. 7B-C). Additionally, enhanced migratory abilities were compromised upon exposure to the STAT3 inhibitor (Fig. 7D). Taken together, these data indicated that SNF5 knockdown led to STAT3 activation, which further promoted cell proliferation and enhanced the migration of BC cells.

### SNF5 expression is associated with differential chemotherapeutic response in BC cells

**Fig. 8:**
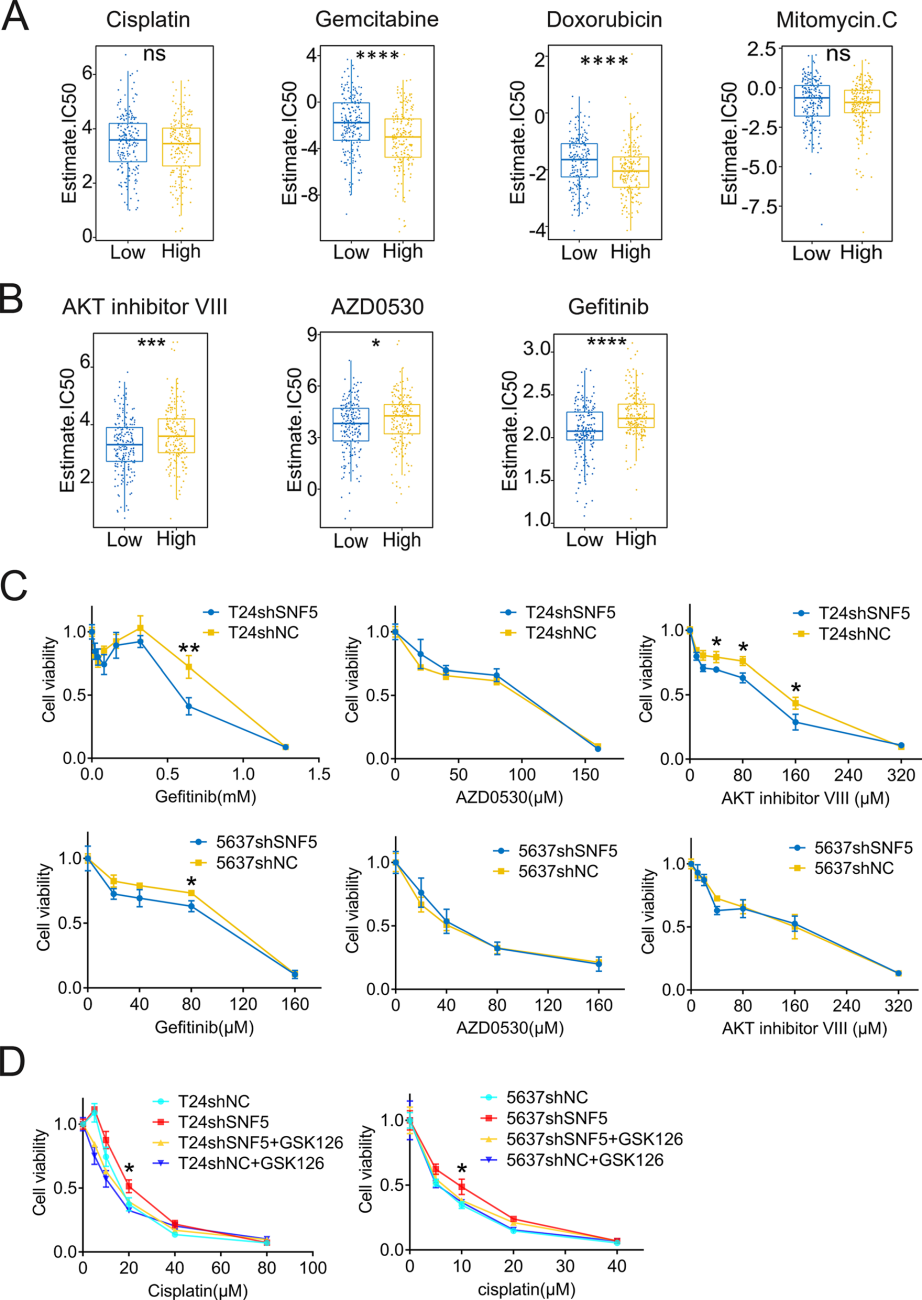
Putative chemotherapeutic responses in BC. **A** The estimated IC_50_ for FDA-approved drugs, including cisplatin, gemcitabine, doxorubicin and mitomycin, in BC patients in TCGA. **B** The estimated IC50 of AKT inhibitor VIII, AZD-0530 and Gefitinib in BC patients and **C** Validation of findings in drug resistance assays. **D** Drug resistance assay was performed to determine the viabilities of the indicated cells exposed to cisplatin with or without GSK126, an EZH2 inhibitor. *p < 0.05, **p < 0.01, ***p < 0.001, ****p < 0.0001

**Fig. 9:**
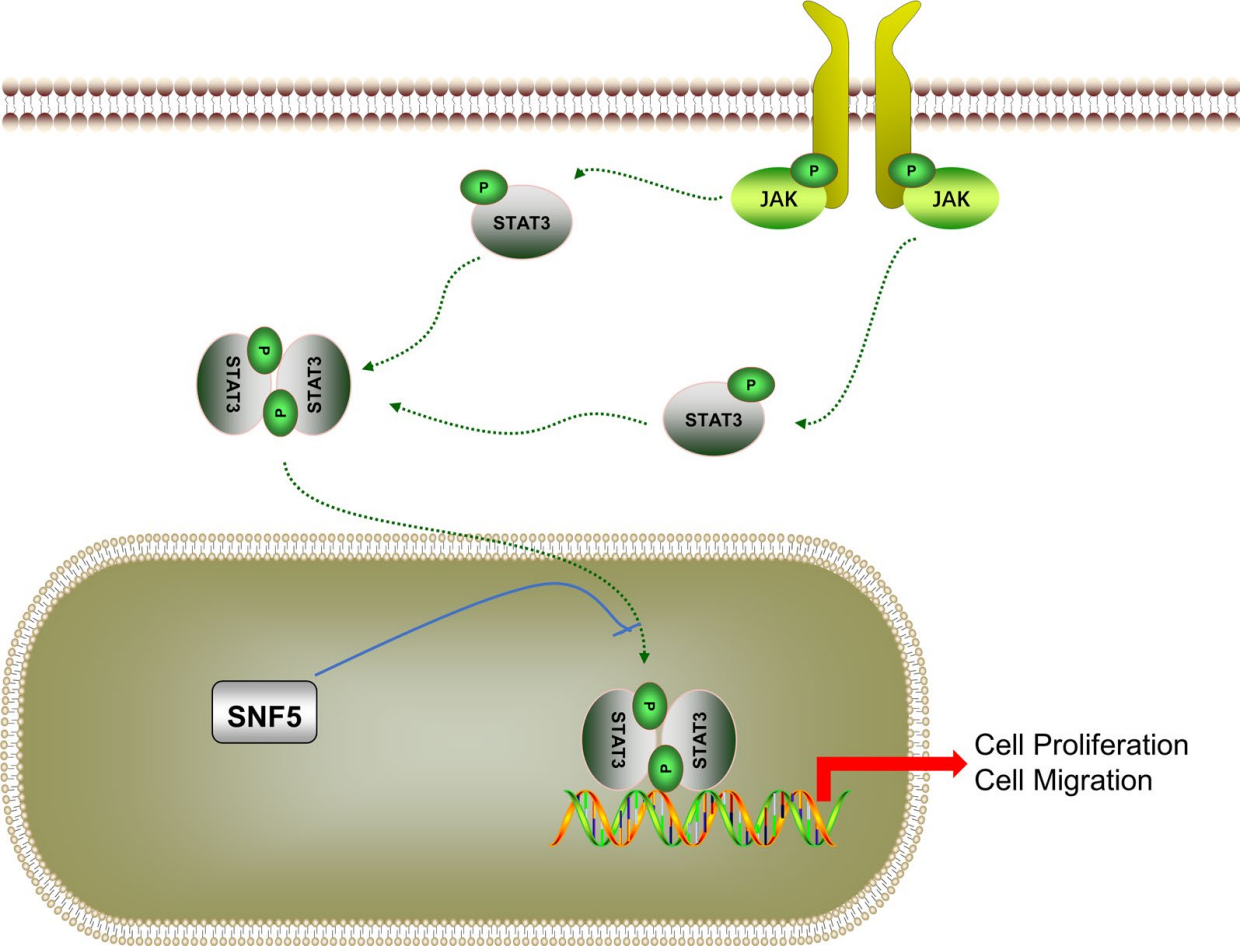
Schematic representation of the proposed mechanism, where low SNF5 expression facilitates cell proliferation and migration by STAT3 activation

Chemotherapy is the mainstay treatment strategy in advanced malignant tumors. Therefore, we initially evaluated the response of BC patients with low-SNF5 and high- expression to the first-line therapeutic drugs. Thereby, a predictive model was established based on the GDSC cell line datasets. Next, we calculated the predicted IC_50_ for the patients in TCGA database using this model to screen candidate drugs based on the SNF5 expression. The estimated IC_50_ of gemcitabine and doxorubicin were significantly higher in the SNF5 low expression than those in the SNF5 high-expression group, which indicated that chemoresistance occurred in patients with low SNF5 expression. Although no significant intergroup differences were observed for cisplatin and mitomycin C treatment in BC, a trend emerged which indicated that patients low SNF5 expression could exhibit increased resistance to these drugs (Figs. 8A and 9).

Moreover, a significantly lower estimated IC_50_ in the SNF5 low- expression group than those in the high-expression group were obtained for some inhibitors, including gefitinib, AZD0530, and AKT inhibitor VIII. Thus, low SNF5 expression could indicate increased sensitivity to these inhibitors (Fig. 8B). Subsequently, we validated the effects of these three drugs on the established cells. The results showed that attenuated SNF5 expression conferred greater sensitivity to gefitinib in both T24- and 5637-SNF5 knockdown cells (Fig. 8C). However, the difference in sensitivity to AKT inhibitor VIII was only observed in T24 SNF5 depleted cells and no significant differences to AZD0530 were observed in SNF5-depleted 5637 and T24 cells.

It is well recognized that EZH2, the catalytic subunit of the PRC complex, antagonizes SNF5 [27]. Given the antagonism between the two molecules, we tested whether the mutually antagonistic effect could sensitize BC cells towards chemotherapeutic drugs. We observed that SNF5 depletion significantly contributed to cisplatin resistance in BC cells (Fig. 8D), which was consistent with the abovementioned computational identification. Notably, GSK126, an EZH2 inhibitor, could impair the resistances of T24shSNF5 and 5637shSNF5 cells against cisplatin.

Collectively, the abovementioned results suggested that SNF5 expression may facilitate decision-making for chemotherapy, thereby guiding the development of individualized treatment regimens for BC patients.

## Discussion

In this study, to the best of our knowledge, for the first time, we identified the function of SNF5 in BC. SNF5 expression was significantly downregulated in BC patients with lymphatic metastasis, and it conferred poor clinical outcomes in BC patients. Functional assays demonstrated that attenuation of SNF5 expression facilitated cell proliferation, both in vitro and in vivo, and enhanced cell migration. Our findings suggested that diminished SNF5 levels may be implicated in STAT3 activation and their cross-talk could promote cell proliferation and migration. Importantly, computational identification and drug-sensitivity experiments provided valuable clues for therapeutic approaches tailored for BC patients based on their SNF5 expression.

Interest in SNF5, the core member of the SWI/SNF complex, initially arose from its frequent inactivation by biallelic mutations in 95% of MRT cases, a highly aggressive and lethal cancer type [9, 28]. Subsequent studies identified that SNF5 deletion occurring due to heterozygous deletions is quite common in other cancers, such as chronic myeloid leukemia and melanoma [29, 30]. Previous studies indicated that SNF5 aberrations are restricted to MRT and there are rarely any mutations in solid tumors [31]. Indeed, the mutation rate of SNF5 was only 4% in BC in TCGA dataset (data not shown), which decreased the likelihood of mutation-induced low SNF5 expression in BC. Moreover, Stachowiak et al. found a marked decrease in SNF5 protein levels in BC specimens [32], but a higher SNF5 mRNA level in BC, which suggested that post-transcriptional mechanisms may be involved in the attenuation of SNF5 expression in BC.

Earlier studies have demonstrated that SNF5 is very closely related to the prognosis of several cancer tumors. We for the first-time report that low SNF5 expression is significantly associated with poor prognosis in BC. This result is in line with previous findings in melanoma, hepatocellular carcinoma and skull base chordoma [30, 33] and further confirmed the tumor-suppressor role of SNF5 in BC. Dysregulated cell cycle is a key driver of uncontrolled cell proliferation in cancer. In this study, SNF5-induced accelerated cell proliferation was in line with previous findings, which have indicated that SNF5 loss results in cell cycle progression and enhanced cell proliferation [34]. Moreover, in vivo data suggested that cyclin D1 is a critical down-stream regulator of carcinogenesis in the absence of SNF5 [35]. Interestingly, cyclin D1 is also downstream of STAT3. Apoptosis is another determinant for tumor growth. In gastric carcinoma, overexpressing SNF5 in cells induce apoptosis by inhibiting Bcl-2 and upregulating Bax [36]. Choudhari et.al show that deactivation of STAT3 can promote apoptosis in hepatocellular carcinoma [37]. However, IHC staining for cleaved caspase 3 appeared to be inconsistent with the above findings. This contradiction suggested the involvement of other signaling pathways in apoptosis upon SNF5 downregulation in BC.

The correlation between the epithelial-mesenchymal transition (EMT) pathway and tumor metastasis has been well established. Compelling evidence demonstrates that STAT3 impacts the invasion and migration of cancer cells via EMT. Furthermore, we found that enhanced migration via EMT regulated by SNF5 depletion aligned with previous findings that suggset high migratory abilities in MRT cells having deletion mutation in SNF5 [38]. Further studies, informed by this work, are needed to elucidate whether SNF5 has an impact on cell invasion in BC. However, we found no significant changes in SNF5-overexpressing T24 cells. This suggested that SNF5, a highly conserved gene, may be present sufficiently enough to exert its suppressive functions on oncogenic signaling pathways in BC.

In our study, the evidence that the aggressiveness of SNF5-depleted BC cells was compromised by STAT blockade, for the first time, provided a critical link between SNF5 and JAK/STAT signaling pathway in malignant phenotypes of BC. There are two possible modes for interaction between SNF5 and STAT3. A recent study shows that SNF5 directly binds to the oncogene, MYC, and impedes target gene recognition by MYC [13]. This finding endows the possibility that the STAT3 signaling pathway is inhibited upon SNF5 binding in BC. Another possible explanation revolves around the indirect mechanism by which SNF5 may regulate the inhibitory upstream pathway of STAT3.

Notably, AKT inhibitor VIII, gefitinib, AZD-0530, and GSK126, could also be preferentially considered for patients with low SNF5 expression. Findings on the activation of AKT in various cancers, especially in SNF5-deficent MRT, are in line with the significant response sensitivity to AKT inhibitor VIII observed in the SNF5-knockdown T24 cells. A phospho-proteomic analysis identified phosphorylation of EGFR in SNF5 deficient cells and found that EGFR inhibition could exert better therapeutic effects in SNF5 deficient cells [39]. Gefitinib, a selective EGFR inhibitor approved by the FDA for the treatment of lung cancer, consistently showed greater sensitivity in SNF5 knockdown BC cells. Currently, a randomized phase III trial is being conducted to investigate whether gefitinib exerts synergistic effects with Bacille Calmette-Guérin (BCG) immunotherapy in high-risk BC patients (Clinical Trial Identifier: NCT00352079). Src, together with EGFR, is involved in tumor development. AZD0530 (saracatinib), a potent inhibitor of Src kinase, can inhibit metastasis in an in vivo model of BC [40]. However, earlier investigations have shown that Src activity had no significant effect on tumor cell growth [41, 42], which helps in explaining the lack of significant difference in drug response for AZD-0530.

The antagonism between SNF5 and EZH2 has been well documented [27], however, the EZH2 inhibitors are not included in pRRophetic R package. Therefore, we investigated whether GSK126, an inhibitor of EZH2, could provide additional benefit over the use of cisplatin alone. Interestingly, GSK126 could sensitize SNF5-depleted cells to cisplatin. Overall, the abovementioned findings provide a basis for the potential clinical application of EGFR-targeted chemotherapy or cisplatin plus EZH2 inhibitor regimens in BC based on SNF5 expression.

In summary, for the first time, we identified the functions of SNF5 and found an association between SNF5 and STAT3 in BC biological features, thereby, enhancing the understanding of mechanisms underlying BC progression. Importantly, the findings may provide valuable clues for the development of therapeutic approaches individually customized for BC patients based on their SNF5 expression in the future.

## Supplementary Information

**Additional file 1: Table S1.** The clinical characteristics of BC patients in TCGA.

**Additional file 2: Table S2.** The clinical characteristics of BC patients in GSE13507 cohort.

**Additional file 3: Table S3.** The associations between clinicopathological variables and the expression of SNF5 in TCGA.

**Additional file 4: Fig. S1** Efficiency of SNF5 knockdown in BC cells was verified by western blotting.

**Additional file 5: Fig. S2** The effects of SNF5 overexpression in T24 cells on proliferation and migration **A** The efficiency of SNF5 overexpression in BC cells was verified by western blotting. **B** CCK8 and colony formation assays were performed to evaluate the proliferative ability of T24 cells. **C** Wound healing assays (100X) and **D** Transwell assays in the indicated cells (200X).

## Data Availability

The datasets generated during and/or analyzed during the current study are available from the corresponding author on reasonable request.

## Abbreviations

BC: Bladder cancer
TCGA: The Cancer Genome Atlas
GEO: Gene Expression Omnibus
GDSC: Genomics of drug Sensitivity in Cancer
GSEA: Gene Set Enrichment Analysis
ROC: Receiver operating characteristic
MRT: Malignant rhabdoid tumors
