# Correction to: Attenuated expression of SNF5 facilitates progression of bladder cancer via STAT3 activation

**DOI:** 10.1186/s12935-021-02436-3

**Published:** 2022-01-14

**Authors:** Hua Ding, Yaqin Huang, Jiazhong Shi, Liwei Wang, Sha Liu, Baixiong Zhao, Yuting Liu, Jin Yang, Zhiwen Chen

**Affiliations:** 1 Department of Urology, Southwest Hospital, Third Military Medical University (Army Medical University), Chongqing, 400038 China; 2 Department of Cell Biology, Third Military Medical University (Army Medical University), Chongqing, 400038 China; 3 Unit 32357 of People’s Liberation Army, Pujiang, 611630 China

## Correction to: Cancer Cell International (2021) 21:655 10.1186/s12935-021-02363-3

In this article [1], the author would like to update the Funding section as below and the description in Fig. 7 has been added with this correction.

1. Funding section should be: This study was supported by the National Natural Science Foundation of China (Grants 81772738, 81572772, 81602250) and Key Talents Support Plan of Army Medical University (2019, 410301053410).
2. Figure 7 legend should be added: *p < 0.05, **p < 0.01, ***p < 0.001.
