# Supplementary material for: Attenuated expression of SNF5 facilitates progression of bladder cancer via STAT3 activation
Source: Cancer Cell Int. 2021 Dec 7;21:655. doi: 10.1186/s12935-021-02363-3 (PMC8650342; doi:10.1186/s12935-021-02363-3)
Supplement: Supplementary file 1 — Additional file 1: Table S1. The clinical characteristics of BC patients in TCGA. [file 12935_2021_2363_MOESM1_ESM.docx]

**Table S1.** **The clinical characteristics of BC patients in TCGA.**

| Clinical characteristics |  | N | (%) |
| --- | --- | --- | --- |
| Age (years) | >=65 | 261 | 63.3 |
|  | <65 | 151 | 36.7 |
| Gender | Male | 304 | 73.8 |
|  | Female | 108 | 26.2 |
| T stage | T0 | 1 | 0.2 |
|  | T1 | 3 | 0.7 |
|  | T2 | 120 | 29.1 |
|  | T3 | 196 | 47.7 |
|  | T4 | 59 | 14.3 |
|  | Tx | 1 | 0.2 |
|  | Not reported | 32 | 7.8 |
| N stage | N0 | 239 | 58.0 |
|  | N1 | 47 | 11.4 |
|  | N2 | 76 | 18.4 |
|  | N3 | 8 | 1.9 |
|  | Nx | 36 | 8.7 |
|  | Not reported | 6 | 1.4 |
| M stage | M0 | 196 | 47.6 |
|  | M1 | 11 | 2.7 |
|  | Mx | 202 | 49.0 |
|  | Not reported | 3 | 0.7 |
| Stage | I | 2 | 0.5 |
|  | II | 131 | 31.8 |
|  | III | 141 | 34.2 |
|  | IV | 136 | 33 |
|  | Not reported | 2 | 0.5 |
| Grade | Low | 21 | 5.1 |
|  | High | 388 | 94.2 |
|  | Not reported | 3 | 0.7 |
