# Supplementary material for: Attenuated expression of SNF5 facilitates progression of bladder cancer via STAT3 activation
Source: Cancer Cell Int. 2021 Dec 7;21:655. doi: 10.1186/s12935-021-02363-3 (PMC8650342; doi:10.1186/s12935-021-02363-3)
Supplement: Supplementary file 2 — Additional file 2: Table S2. The clinical characteristics of BC patients in GSE13507 cohort. [file 12935_2021_2363_MOESM2_ESM.docx]

**Table S2.** **The clinical characteristics of BC patients in GSE13507 cohort.**

| Clinical characteristics |  | N | (%) |
| --- | --- | --- | --- |
| Age (years) | >=65 | 96 | 58.2 |
|  | <65 | 69 |  |
| Gender | Male | 135 | 81.8 |
|  | Female | 30 |  |
| T stage | Ta | 24 | 14.5 |
|  | T1 | 75 | 45.5 |
|  | T2 | 36 | 21.8 |
|  | T3 | 19 | 11.5 |
|  | T4 | 11 | 6.7 |
| N stage | N0 | 150 | 90.9 |
|  | N1 | 7 | 4.2 |
|  | N2 | 6 | 3.6 |
|  | N3 | 1 | 0.6 |
|  | Nx | 1 | 0.6 |
| M stage | M0 | 158 | 95.8 |
|  | M1 | 7 | 4.2 |
