# Supplementary material for: Attenuated expression of SNF5 facilitates progression of bladder cancer via STAT3 activation
Source: Cancer Cell Int. 2021 Dec 7;21:655. doi: 10.1186/s12935-021-02363-3 (PMC8650342; doi:10.1186/s12935-021-02363-3)
Supplement: Supplementary file 3 — Additional file 3: Table S3. The associations between clinicopathological variables and the expression of SNF5 in TCGA. [file 12935_2021_2363_MOESM3_ESM.docx]

**Table S3. The associations between clinicopathological variables and the expression of SNF5 in TCGA.**

| Characteristic | Low expression of SNF5 | High expression of SNF5 | p |
| --- | --- | --- | --- |
| n | 207 | 207 |  |
| T stage, n (%) |  |  | 0.692 |
| T1 | 3 (0.8%) | 2 (0.5%) |  |
| T2 | 54 (14.2%) | 65 (17.1%) |  |
| T3 | 100 (26.3%) | 96 (25.3%) |  |
| T4 | 32 (8.4%) | 28 (7.4%) |  |
| N stage, n (%) |  |  | 0.384 |
| Absent (N0) | 115 (31.1%) | 124 (33.5%) |  |
| Present (N1+N2+N3) | 70 (18.9%) | 61 (16.5%) |  |
| M stage, n (%) |  |  | 0.727 |
| M0 | 94 (44.1%) | 108 (50.7%) |  |
| M1 | 4 (1.9%) | 7 (3.3%) |  |
| Gender, n (%) |  |  | 0.264 |
| Female | 60 (14.5%) | 49 (11.8%) |  |
| Male | 147 (35.5%) | 158 (38.2%) |  |
| Age, n (%) |  |  | 0.921 |
| <=70 | 118 (28.5%) | 116 (28%) |  |
| >70 | 89 (21.5%) | 91 (22%) |  |
| Histologic grade, n (%) |  |  | 0.068 |
| High Grade | 201 (48.9%) | 189 (46%) |  |
| Low Grade | 6 (1.5%) | 15 (3.6%) |  |
| AJCC stage, n (%) |  |  | 0.521 |
| Stage I | 3 (0.7%) | 1 (0.2%) |  |
| Stage II | 60 (14.6%) | 70 (17%) |  |
| Stage III | 72 (17.5%) | 70 (17%) |  |
| Stage IV | 72 (17.5%) | 64 (15.5%) |  |
