# Supplementary figures and images for: Attenuated expression of SNF5 facilitates progression of bladder cancer via STAT3 activation
Source: Cancer Cell Int. 2021 Dec 7;21:655. doi: 10.1186/s12935-021-02363-3 (PMC8650342; doi:10.1186/s12935-021-02363-3)

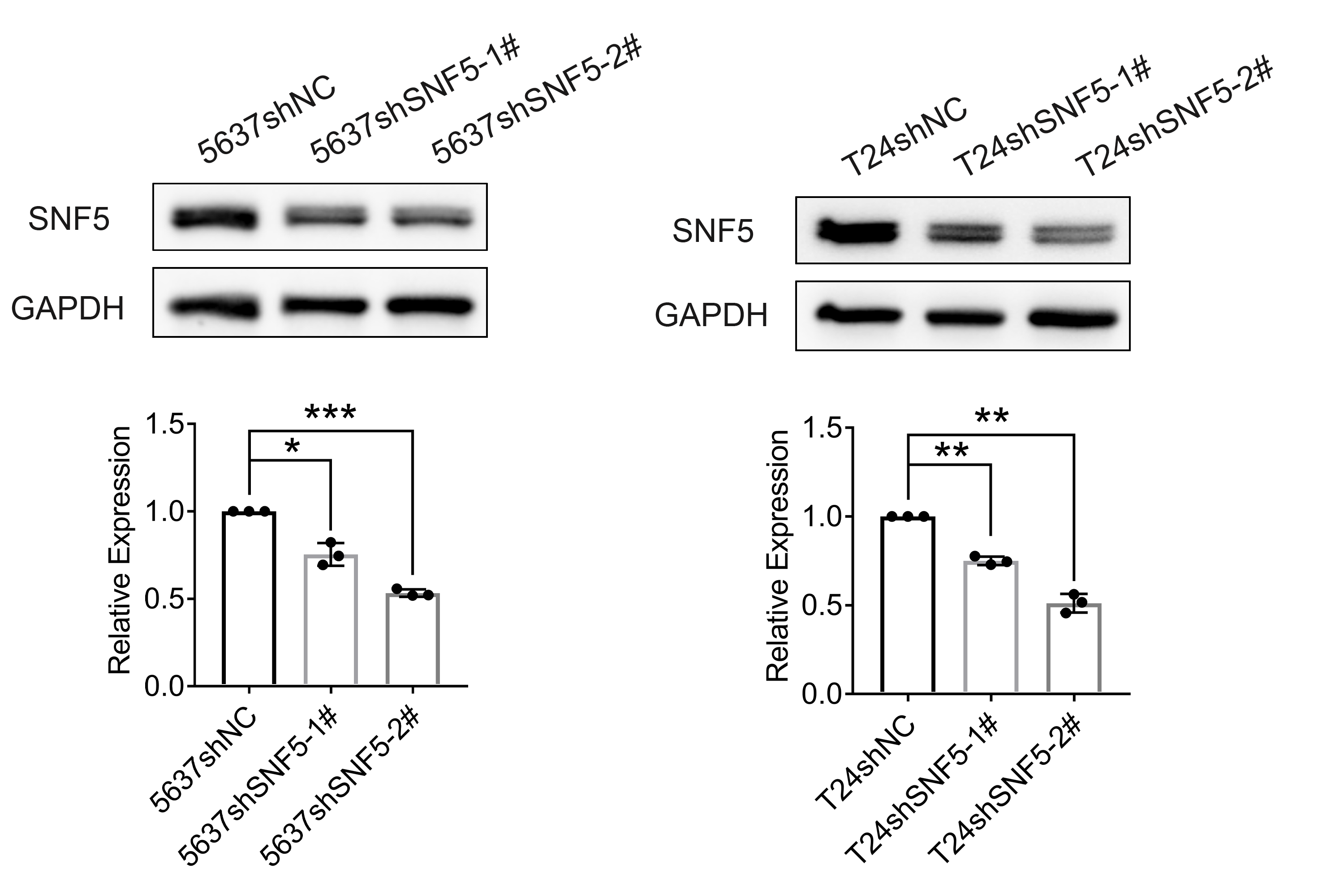

Supplement: Supplementary file 4 — Additional file 4: Fig. S1 Efficiency of SNF5 knockdown in BC cells was verified by western blotting. [file 12935_2021_2363_MOESM4_ESM.tif]

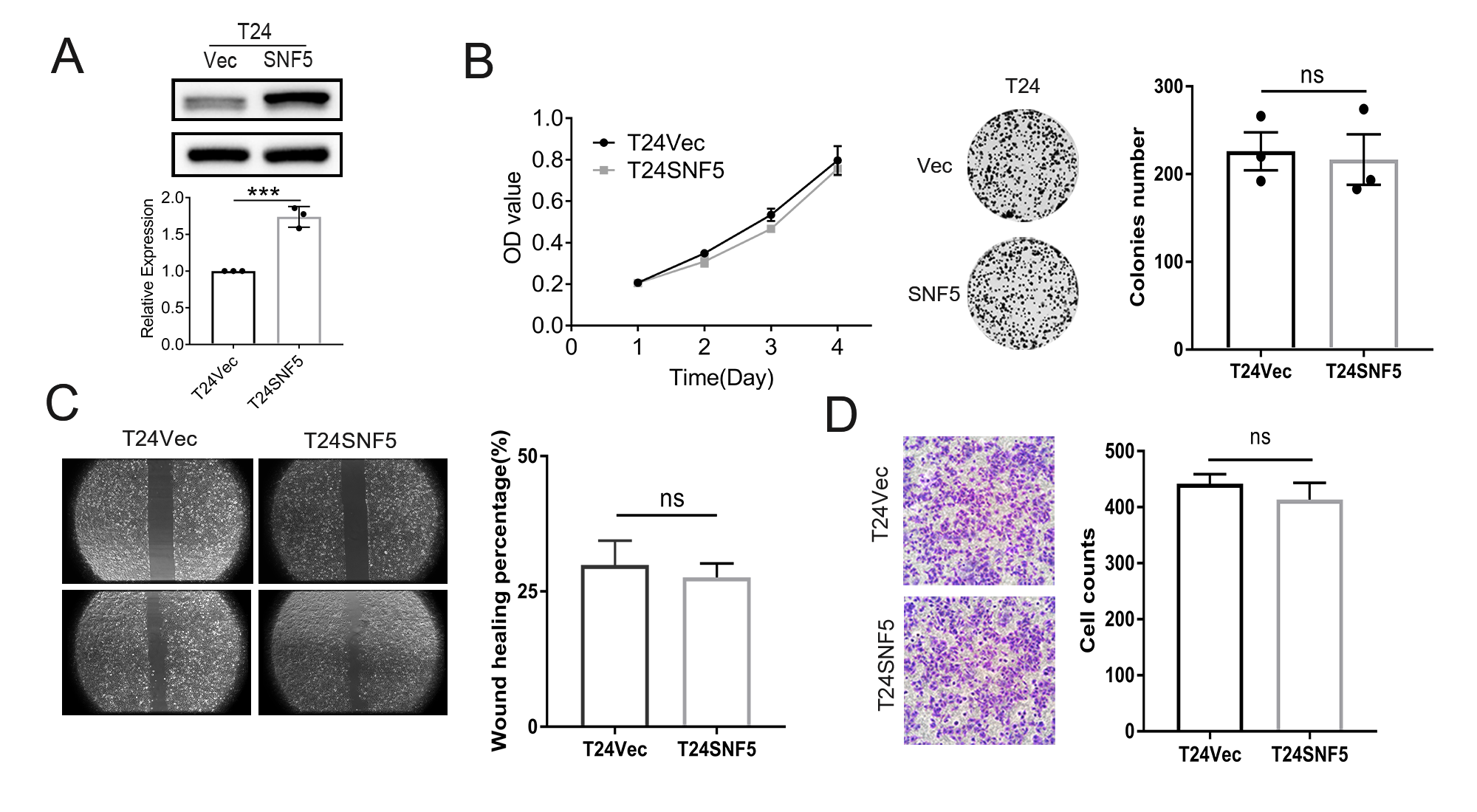

Supplement: Supplementary file 5 — Additional file 5: Fig. S2 The effects of SNF5 overexpression in T24 cells on proliferation and migration A The efficiency of SNF5 overexpression in BC cells was verified by western blotting. B CCK8 and colony formation assays were performed to evaluate the proliferative ability of T24 cells. C Wound healing assays (100X) and D Transwell assays in the indicated cells (200X). [file 12935_2021_2363_MOESM5_ESM.tif]
